# A process model of psychological acceptance in technology-based service innovation: a symbolic interactionism perspective

**DOI:** 10.3389/fpsyg.2026.1805811

**Published:** 2026-09-18

**Authors:** Wen-Hong Chiu, Chi-Quyen Luong, Hui-Ru Chi

**Affiliations:** 1 School of Law and Public Affairs, Jiaying University, Meizhou, China; 2 School of Economics and Management, Hunan Institute of Science and Engineering, Yongzhou, China

**Keywords:** diffusion of innovation, evidence-based medicine, process model, symbolic interactionism, technology-based service innovation

## Abstract

As digital technologies become increasingly embedded in service systems, the diffusion of technology-based service innovations is often gradual and contested rather than automatic. Prior research has provided robust explanations for early-stage technology evaluation by treating adoption as a discrete decision or momentary experience. However, it provides limited insight into the longer-term process through which user acceptance gradually develops through ongoing use. Drawing on symbolic interactionism, this study reconceptualizes diffusion of technology-based service innovation as a process of symbolic meaning construction unfolding through ongoing interaction and use. Based on a qualitative study of an evidence-based medicine (EBM) system implemented in hospitals in Taiwan region, the study develops an integrative process model that explains how users' relationships with a technology evolve from initial unfamiliarity to routine integration in professional practice. The findings show that diffusion advances not only through positive functional evaluation but also through interpretive alignment and the gradual internalization of shared meanings through everyday service interactions. Diffusion begins with users moving from initial evaluative meanings, which are progressively shaped into collectively negotiated and socially validated interpretations, ultimately leading to the internalization of shared meanings and their circulation in use. By conceptualizing diffusion as a process of symbolic meaning construction in use, the study reveals the psychological and interpretive dynamics through which user acceptance develops beyond initial evaluation.

## 1 Introduction

The evolution of technology-based services has increasingly shifted from isolated tools for efficiency enhancement toward integrated service systems that reshape work practices, coordination, and user roles (Abid et al., 2025; Burton et al., 2024). Within this broader transformation, the emerging digital technologies are increasingly embedded in service processes, extending beyond automation or information processing to influence decision-making and professional practice (Akter et al., 2023; Kim, 2023; Manser Payne et al., 2021). However, the diffusion of technology-based service innovations in practice has been characterized by a gradual and contested adoption process. Empirical evidence from large-scale AI medical initiatives suggests that technological sophistication does not automatically translate into user acceptance or effective service innovation. For example, AI-driven clinical decision support systems (AI-CDSS) have been widely applied in public hospitals as part of smart healthcare and digital governance initiatives. Although these systems are ostensibly designed to support clinical decision-making, their implementation may shape how healthcare professionals interpret technology in relation to standardization, supervision, and performance monitoring (Elgin and Elgin, 2024; Pardoux and Kerasidou, 2025; Zhao et al., 2025). Such experiences suggest that behavioral adoption and psychological acceptance may not always develop in parallel. Psychological acceptance concerns how individual users come to understand and accept the technology (AlQudah et al., 2021; Chong et al., 2022). It differs from behavioral adoption, which refers to the decision or act of using the technology, and from continued use, which concerns sustained behavioral engagement after adoption (Yan et al., 2021; Zhou et al., 2024). The primary level of analysis is the individual user, while collective diffusion emerges through the development and spread of meanings and practices across users within professional contexts. This distinction highlights the need to explore how psychological acceptance develops over time and contributes to broader diffusion.

Accordingly, scholarly attention has increasingly turned to diffusion of innovation (DOI), technology acceptance model (TAM), human–computer interaction (HCI), and user experience (UX) research as core perspectives for explaining how users engage with and appropriate technology-based service innovations (Rogers, 2003; Davis, 1989; Venkatesh et al., 2003). Despite their extensive use, these theoretical perspectives share several important limitations when applied to the diffusion of technology-based service innovation. First, DOI and TAM primarily conceptualize adoption as an individual-level decision driven by perceived attributes, attitudes, or behavioral intentions at particular points in time (de Brauwer, 2024; Vargo et al., 2020; Ibrahim et al., 2025). These frameworks privilege the moment of adoption, providing limited insight into how psychological acceptance evolves through continued use and experience. Second, HCI and UX research, while attentive to user–technology interaction, predominantly adopt a design- and experience-centric lens, emphasizing usability and localized interactional episodes (Berni et al., 2023; Martinelli et al., 2024; Lemmo et al., 2025). Consequently, they offer a limited insight into how user acceptance changes psychologically over time.

In the context of technology-based services, innovation diffusion is not merely a cognitive decision but an ongoing process in which users gradually interpret, negotiate, and reconstruct its meaning through everyday service experiences and social interactions (Bach et al., 2024; Elmaresa, 2025; Mele et al., 2019; Mesenhöller, 2025). From a symbolic interactionist perspective, users' engagement with technology is continuously mediated through symbols and meanings that are actively interpreted and reinterpreted in use. Rather than responding to technological innovations solely based on functional attributes, users engage in sensemaking through interpretive processes embedded in everyday service practices, with meanings continuously unfolding and evolving across stages of interaction and use (Chen et al., 2020; Zhong et al., 2025). Such symbolic interactions shape not only patterns of technology use but also how users' perceptions, experiences, and evaluations of an innovation evolve (Almokdad et al., 2025). As technology-based service innovations become increasingly embedded in complex service systems, innovation diffusion extends beyond a one-off or purely individual adoption decision. In this sense, innovation diffusion in technology-based service contexts unfolds as an ongoing process in which users continuously construct and stabilize meaning through repeated interaction and use (Vargo et al., 2020; Trischler et al., 2020). Unlike DOI, TAM, and HCI/UX perspectives, which primarily explain adoption decisions and user-level interaction experiences (de Brauwer, 2024; Berni et al., 2023; Martinelli et al., 2024), symbolic interactionism provides a theoretical lens for understanding how these meanings are interpreted, negotiated, and collectively sustained through social interaction (Mørch et al., 2024; Chen et al., 2020). Therefore, a symbolic interactionist perspective provides a critical theoretical foundation for revealing how innovation diffusion unfolds as a process of constructing symbolic meaning.

Accordingly, this study aims to explain how psychological acceptance of technology-based service innovation evolves through ongoing symbolic interaction and meaning construction during the diffusion process. Grounded in symbolic interactionism, it reconceptualizes diffusion as a process of symbolic meaning construction rather than a linear trajectory of adoption and develops a process model of psychological acceptance. Empirically, the study examines the implementation of an evidence-based medicine (EBM) system in hospitals in Taiwan region as a representative case of technology-based service innovation. EBM systems represent a knowledge-intensive form of technology-based service innovation that seeks to translate standardized medical evidence into clinical decision-making (Scalise, 2005). Because EBM systems are introduced into established professional routines, their diffusion directly engages physicians' evolving interpretations of expertise, autonomy, and clinical judgment in everyday clinical work. This makes EBM systems not merely a technical innovation, but a symbolically charged form of technology-based service innovation.

## 2 Literature review

### 2.1 Adoption-centered perspectives on diffusion of innovation and technology acceptance

The diffusion of innovation (DOI) theory conceptualizes innovation adoption as a dynamic communication process in which an innovation is disseminated through specific channels among members of a social system (Rogers, 2003; Carreiro and Oliveira, 2019). DOI primarily explains adoption by focusing on individuals' perceptions of innovation attributes, such as relative advantage, compatibility, and complexity, and how these perceptions shape adoption rates across a population (Bernuy et al., 2025; Liu et al., 2023). Although DOI acknowledges the role of social systems and communication channels, its analytical emphasis ultimately lies in explaining how individual evaluative judgments toward innovation characteristics translate into adoption outcomes (de Brauwer, 2024; Min et al., 2019; Vargo et al., 2020). Similarly, the technology acceptance model (TAM) has been extensively used to explain users' acceptance of information technologies by emphasizing perceived usefulness and perceived ease of use as key antecedents of attitudes, behavioral intention, and actual use (Al-Adwan et al., 2023; Davis, 1989; Song et al., 2025).

However, both frameworks share a critical limitation, that is, they conceptualize acceptance as a static, outcome-oriented decision made at a specific point in time. They prioritize the moment of adoption, treating it as the endpoint of the process, rather than the beginning of an ongoing journey of interpretation and experience (de Brauwer, 2024; Ibrahim et al., 2025; Vargo et al., 2020). By focusing on stable individual evaluations and attitudes, they fail to account for the dynamic, staged process through which users' psychological acceptance evolves and is renegotiated in the context of everyday use. Consequently, these models leave a significant gap in understanding the internal evolutionary logic of users' psychological changes over time.

### 2.2 Interaction- and experience-centered perspectives on human–computer interaction and user experience

Human–computer interaction (HCI) primarily focuses on how humans interact with computing systems and how such interactions can be designed to enhance usability, effectiveness, and efficiency (Søgaard Neilsen and Wilson, 2019; Zhao et al., 2024; Zhou et al., 2025). Traditional HCI studies rely heavily on laboratory-based cognitive experiments, prioritizing functional interaction and task performance evaluation (Gulati et al., 2024; Iarygina et al., 2025). To address the insufficiency of purely functional assessment, contemporary HCI scholarship has gradually incorporated user experience (UX) perspectives, recognizing that human–technology interaction cannot be adequately understood or designed solely in terms of functional performance (Chen and Zhou, 2025; Lu et al., 2025). UX research complements conventional HCI by focusing on users' subjective, affective, and situational perceptions arising from interaction with products, services, or systems (Huo et al., 2023; Okonkwo, 2024; Lu et al., 2025). It integrates both pragmatic usability and hedonic emotional experiences, extending HCI research beyond instrumental task outcomes to users' holistic lived interaction experiences (Mariano et al., 2024; Mortazavi et al., 2023; Hirsch et al., 2024; Hughes and Karwowski, 2025).

Despite these developments, HCI and UX research remain fundamentally oriented toward interaction episodes and individualized experiences. The dominant analytical focus is placed on usability metrics, interface features, affective responses, and short-term experiential outcomes (Ntoa, 2025; Zhou et al., 2025; Snow et al., 2025). Even socially oriented concepts such as parasocial interaction and social responses to communication technologies primarily focus on how users initially attribute human-like qualities to technological artifacts, rather than how deeper social dynamics operate in this process (Liu, 2025; Hirsch et al., 2024; Qi et al., 2025). Moreover, technological meanings are not fixed but are continuously negotiated, stabilized, and reinterpreted through ongoing social and technological interactions embedded in everyday practice (Bengtsson and Johansson, 2022; Markham, 2024). Consequently, existing HCI and UX research predominantly employs rational cognitive models and overlooks the social symbolic interaction mechanism that underpins users' evolving psychological states.

### 2.3 Symbolic interactionism and the meaning dynamics of technology adoption

Symbolic interactionism was initially developed by Mead (1934) and formalized by Blumer (1969) as a sociological perspective that explains social action as grounded in the interpretation of symbols. Its core premise is that objects, technologies, and events do not possess inherent meanings. Instead, meaning emerges through ongoing interactions and interpretations among individuals within specific social contexts (Ali et al., 2022; Zhong et al., 2025). Without interaction, meaning does not exist (Li, 2025).

Symbolic interactionism emphasizes that meanings are not fixed but continuously constructed, negotiated, and revised through social interaction (Mørch et al., 2024; Li, 2025). Individuals act toward objects based on the meanings they ascribe to them, which are socially generated through interaction and subsequently modified through interpretive processes over time (Blumer, 2004; Mørch et al., 2024; Peng, 2024). This theoretical perspective has been increasingly applied to the study of technological change, as digital technologies function not only as functional tools but also as symbolic objects embedded with social meanings (Chen et al., 2020; Liu Y., 2025). Accordingly, symbolic interactionism provides a powerful framework for examining technology-based service innovation, as it highlights how users assign meanings to new technologies, express identity and status through technology use, and continuously reinterpret technological systems through ongoing interaction (Chen et al., 2020; Qi et al., 2025). At its core, this perspective conceptualizes technology diffusion as an ongoing process through which users continuously construct, interpret, and revise technological meanings through social interaction rather than as a one-time cognitive adoption decision (Bengtsson and Johansson, 2022; Markham, 2024).

Despite its relevance for understanding dynamic meaning construction, existing applications of symbolic interactionism have largely focused on technological artifacts, organizational settings, or individual technology use (Nysveen et al., 2020; Silberer et al., 2023; Sjödin et al., 2020). Comparatively limited attention has been paid to technology-based service innovations. These contexts are characterized by sustained interaction and evolving symbolic meanings. In such settings, psychological acceptance emerges through ongoing processes of meaning construction rather than discrete adoption decisions.

## 3 Methods

This study adopts an exploratory case study approach. The method is particularly suitable for investigating phenomena that remain theoretically underdeveloped and insufficiently explained by existing frameworks (Yin, 2014; Eisenhardt and Graebner, 2007). This condition applies to users' psychological acceptance of technology-based service innovation. Besides, the study aims to explore how psychological acceptance emerges and develops through users' ongoing engagement with technological service innovation. Exploratory case studies are especially appropriate for addressing “how” and “why” questions in relatively unexplored contexts (Yin, 2014).

In addition, the method is effective for examining complex, multilayered, and dynamic processes that cannot be adequately captured through variable-centered or cross-sectional approaches (Fiss, 2011). It also supports inductive theory building and longitudinal process tracing, enabling the development of novel constructs and process explanations (Ponelis, 2015; Yin, 2014). For these reasons, an exploratory case study was adopted as the primary research method.

### 3.1 Case: EBM service system

The idea of EBM came from clinical epidemiologists at McMaster University in Canada (Haynes, 2002). Cohen et al. (2004) pointed out that the core concepts of EBM originated from applying the principles of epidemiology, to the practice of patient care during the 1970s and 1980s. The purpose of EBM is to integrate the best research evidence, clinical expertise, and patient values into the practice of patient care (Cohen et al., 2004; Haynes, 2002; Sackett et al., 2000). In this study, EBM is examined as a digital service system that physicians encounter, interpret, and integrate into their everyday clinical work. The main function of an EBM server is to provide a medical knowledge database for any linked user (or client). An EBM server basically consists of a knowledge database system, a management system, and medical knowledge sources (Cheng et al., 2009). Once implemented, the EBM system becomes part of physicians' routine work, requiring users to interpret and evaluate its relevance through repeated use. Furthermore, suggested solutions can also be provided to support decision-making in medical treatment.

### 3.2 Research site

The research site, referred to as Hospital A, is a district teaching hospital located in central Taiwan region, established in 1976. Hospital A was selected as the research site because prior involvement in the EBM implementation enabled prolonged engagement and close observation of how users interacted with and made sense of the system over time. The hospital employs approximately 1,600 staff members and operates nearly 1,000 beds. Its core responsibilities include the prevention of infectious diseases, the promotion of public health, and the provision of long-term care services.

Over time, Hospital A has completed the establishment of several specialized medical centers, including the Cardiovascular Medicine Center, the Hepatobiliary Medicine Center, and the Health Management Center. As part of a university–industry collaboration, the consulting engagement related to EBM implementation lasted for 1 year. During this period, multiple consulting activities were undertaken, including advisory meetings, the design and delivery of training programs, and the establishment of organizational structures supporting EBM realization.

Informal consultation meetings were conducted on a weekly basis, while formal meetings and focus group interviews were held approximately once per month. Participants comprised managerial personnel from multiple hospital departments, including eight senior executives, 12 junior executives, and 16 supervisors. In total, 36 mid-level and senior managers participated in the study. In addition, participants provided internal documents to substantiate and contextualize the interview data. Through sustained engagement in the consulting process, the research team developed an in-depth understanding of the organizational context and practices associated with EBM realization at Hospital A.

### 3.3 Data collection

In addition to participant observation conducted during the consulting period, in-depth interviews were conducted to examine physicians' experiences, interpretations, and evolving understandings of the EBM system following its introduction. The interviews were conducted in four stages, with each interview lasting approximately 1 h. The study involved 54 interviews, with some participants being interviewed more than once.

In the initial stage, interviews were conducted with one physician, one pharmacist, and two nursing staff members from Hospital A. As the latter interviews yielded limited insights into EBM realization, subsequent interviews focused exclusively on physicians. Among the interview participants, 10 were physicians, including five from Hospital A, four from a tertiary medical center (Hospital B), and one from a branch of another medical center (Hospital C). Hospital B was included due to its recognized excellence in EBM implementation. It is a top-tier medical center in central Taiwan region, founded in 1896, employing approximately 8,500 staff members and operating more than 3,600 beds. All participating physicians had at least 5 years of experience with EBM and served as EBM team leaders or seeded teachers responsible for promoting EBM within their organizations.

In the second stage, the EBM team leader and three seeded teachers from Hospital A were interviewed to capture experiences of interacting with the EBM system. In the third stage, the four physicians from Hospital B were interviewed again to enhance data triangulation. The emerging themes largely overlapped with earlier findings, indicating theoretical saturation. In the final stage, peer debriefing and interviewee validation were conducted with EBM team leaders from Hospitals B and C, further confirming saturation of key aspects of EBM realization.

Clarification of participant groups and data sources. The 36 managerial participants from Hospital A contributed 43 interview sessions. The four senior executives from Hospital B contributed nine interview sessions, and the senior executive from Hospital C contributed two interview sessions. Together, these participants accounted for 54 interview sessions, with some participants interviewed more than once across the different stages of data collection. The interview data formed the primary empirical basis for construct development, while participant observation, consulting records, internal documents, archival materials, and secondary sources were used primarily for contextualization and triangulation. The participant codes AISE, AIJE, and AIS refer to senior executives, junior executives, and supervisors from Hospital A, respectively. The BISE refers to senior executives from Hospital B, and CIJE refers to the senior executive from Hospital C. OD1 and OD2 denote the two observation periods. These codes indicate participants' organizational roles, while all participants were physicians by professional background. In Table 1, the “No.” column indicates the number of records collected for each data source.

**Table 1:** Overview of data sources, documents, and document codes.

Data sources	**No**.	**Abbreviations**
**Primary data sources**		
Interviews with 8 senior executives of Hospital A (July 2022 to March 2024; average interview length 108 min)	12	AISE 1–8
Interviews with 12 junior executives of Hospital A (July 2022 to October 2023; average interview length 79.3 min)	14	AIJE 1–12
Interviews with 16 supervisors of Hospital A (July 2022 to March 2024; average interview length 56 min)	17	AIS 1–16
Interviews with 4 senior executives of Hospital B (May 2024 to June 2024; average interview length 85.2 min)	9	BISE 1–4
Interviews with 1 senior executives of Hospital C (July 2024; average interview length 76.6 min)	2	CIJE 1
Full participation in the EBM plan of hospital A (July 2022 to June 2023)	27	OD1 1–27
Participant observation of successful realization of EBM in hospital A (July 2023 to March 2024)	15	OD2 1–15
**Archival and secondary data sources**		
Internal documents (including paper data and electronic data; July 2022 to June 2023)	35	ID-D 1–35
Hospital accreditation data system (including data sheet, statistical table, relevant attached file, briefing documents, etc.; July 2022 to June 2023)	44	ADS-D 1–44
EBM project team meeting records (July 2022 to June 2023)	67	MR-D 1–67
EBM project planning documentation (July 2022 to June 2023)	9	PRO-D 1–9
EBM knowledge website (March 2024)	39	WEB-D 1–39
Media coverage about the EBM (July 2024)	17	MC-D 1–17
Popular press articles (July 2022 to Mach 2023)	42	PA-D 1–42
Hospital industry analysis report (October 2023)	11	IR-D 1–11
University lecture about the EBM (August 2023)	2	EBM-LEC
Taiwan Evidence-Based Medicine Association, Taiwan region (July 2023 to February 2024)	26	TWA 1–26
**Total**	**388**	

To enhance trustworthiness, triangulation was applied throughout the research process. Data sources included observations, meetings, focus group interviews, internal documents, follow-up communications with interviewees, and secondary data such as historical records, meeting minutes, and website materials. In total, 388 documents were collected across the consulting period and the four-stage interview process, as summarized in Table 1. Before data collection, all participants were informed of the research purpose, procedures, and the voluntary nature of participation. Verbal informed consent was obtained before interviews, observations, and focus group discussions, and participants were informed of their right to withdraw at any time without adverse consequences. To protect confidentiality, all identifying information was removed from transcripts and field records. The study did not involve sensitive personal information or procedures that could pose physical or psychological harm. All research data were securely stored and used solely for academic research purposes.

### 3.4 Data analysis

Following the Gioia methodology (Gioia et al., 2013), this study adopted an iterative and inductive thematic coding approach for qualitative data analysis. First, independent open coding was performed line by line across interview transcripts, field notes, and other qualitative materials to generate initial descriptive codes rooted in raw data. Subsequently, in accordance with the standard Gioia analytical framework, all first-order codes were systematically reviewed, compared, and grouped into preliminary categories based on semantic relevance and conceptual similarity. Axial coding was then applied to integrate related categories and clarify their internal logical connections, which gradually evolved into second-order themes. Through multiple rounds of iteration, comparison, and revision, these themes were further refined and synthesized into aggregated dimensions, forming the final conceptual constructs of this study.

To ensure coding reliability, ongoing cross-checking and peer debriefing were implemented throughout the analysis. Any inconsistencies were fully discussed until a consensus was reached. The data structure and coding are shown in Table 2, which illustrates the linkage between raw coding data, first-order concepts, second-order themes, and aggregate constructs.

**Table 2:** Data structure and coding.

**Key quote/event**	First-order concept	Second-order theme	Aggregate construct
“*Now, I clearly know how to acquire medical knowledge since the system acts as a learning assistant*”; “*Network becomes my textbooks…my teacher*” (*AISE 1, 90–154*).	EBM platforms serve as learning assistants and instructional resources	Support	Cognitive benefit
“*There are some good and prefiltered databases in some websites*” (*AISE 1, 90–154*).	Availability of high-quality prefiltered databases	Support	Cognitive benefit
“*EBM, just like an assistant, helps me to solve some clinical problems in time*” (*AIJE 3, 50–84*); “*Sometimes I meet unfamiliar clinical problems questioned by some patients, and then I immediately question EBM and it quickly responds*” (*AIS 2, 77–110*).	EBM assists in the timely resolution of clinical problems	Efficiency	Cognitive benefit
“*Some powerful search engines can rank the papers you retrieve… Thus, this saves more time for you*” and “*Since I touch the systematic learning way, I can readily filter papers and efficiently appraise papers*” (*CIJE 1, 62–94*).	Search engines improve the efficiency of literature retrieval and appraisal	Efficiency	Cognitive benefit
“*Even a daily and ordinary issue is investigated by a serious experimental design and subsequently formed as valuable evidence… It really breaks my traditional viewpoints on research*” (*AISE 8, 42–64*).	EBM reshapes traditional understandings of medical research	Effectiveness	Cognitive benefit
“*Most of the published papers in the EBM databases possess a rigorous and complete experimental design. By understanding the experimental steps and protocols, the research result is valuable for supporting my clinical decision*” (*AISE 5, 29–59*); “*I regard EBM as a professional assistant because it is based on a rigorous and systematic methodology*” (*BISE 3, 19–68*).	Rigorous EBM evidence supports clinical decision-making	Effectiveness	Cognitive benefit
“*I think EBM can provide objective and correct solutions for some clinical problems*” (*AIS 9, 120–153*); “*If you refer to EBM papers, your solutions are viewed as more standard and correct*” (*BISE 4, 63–101*).	Gain standard and objective clinical solutions	Effectiveness	Cognitive benefit
“*When I come against unfamiliar clinical problems, the first step I take is to retrieve it*” (*EBM database*), “*This is because the EBM papers have high validity*” (*BISE 4, 63–101*).	Search valid literature for unfamiliar clinical problems	Effectiveness	Cognitive benefit
“*Some databases, for example, Cochrane library, further provide a prefiltered literature*” (*AIJE 10, 82–146*).	Access to pre-filtered evidence-based literature	Effectiveness	Cognitive benefit
“*When my medical knowledge is continuously updated through EBM learning, I actually feel confident in clinical decisions and patient care*” “*Through retrieving the EBM papers, I can follow new evidence and medical trends and hence enhance my professional confidence*” (*AIJE 6, 47–87*).	Enhance professional confidence with updated medical knowledge	Confidence	Psychological benefit
“*I can immediately solve some clinical problems, thanks to EBM learning*” (*AISE 5, 17–39*).	EBM enables immediate resolution of clinical problems	Confidence	Psychological benefit
“*Since the evidence is rigorously derived by researchers, I have more confidence in applying the evidence to my patients*” (*AISE 5, 17–39*).	Increase confidence in using rigorous clinical evidence	Confidence	Psychological benefit
“*I feel so happy to find the solutions to my problems*” (*AIJE 7, 115–129*).	Solving clinical problems generates positive emotions	Enjoyment	Psychological benefit
“*When I browse EBM websites, this is as if I travel in a knowledge palace*” (*AIS 5, 25–58*); “*To my surprise, I find this case interesting and enjoyable!*” (*AISE 2, 63–81*).	EBM knowledge exploration becomes enjoyable and engaging	Enjoyment	Psychological benefit
“*When I come upon any clinical problem, the first step is to retrieve EBM research… In daily work, I cannot do without EBM*”; “*It is necessary for me to use EBM tools since it provides clinical suggestions*”; “*I have a high tendency of usage because I need it*” (*BISE 1, 59–82*).	Develop reliance on EBM for daily clinical work	Attachment	Psychological benefit
“*I cannot do without EBM*” (*AIS 14, 19–46*).	EBM becomes difficult to separate from daily practice	Attachment	Psychological benefit
“*Before the EBM realization, almost every physician had his own opinions and hence we frequently had disputes on some issues*” (*AIS 11, 81–135*).	EBM establishes common interpretive standards among physicians	Shared language	Social meaning
“*We have no idea about the validity of some literature before EBM realization. But now we can reach a consensus by following EBM criteria*” (*AIS 11, 81–135*).	Shared EBM criteria facilitate professional consensus	Shared language	Social meaning
Physicians and clinical teams jointly discuss and establish standardized criteria for evaluating clinical evidence during EBM implementation meetings (OD1 1, 5–27).	EBM criteria are co-developed through collective professional deliberation	Shared language	Social meaning
In multidisciplinary case discussions, physicians with different specialties often present divergent treatment opinions, which are ultimately reconciled by referring to shared EBM guidelines and standardized clinical protocols (PRO-D 2, 4–18).	EBM protocols serve as authoritative reference points for resolving inter-professional disagreements	Shared language	Social meaning
“*EBM also teaches me to focus on patient value*” (*CIJE 1, 38–84*).	Prioritize patient value in clinical practice	Shared value	Social meaning
“*The best evidence is not the best solution for patients*” (*CIJE 1, 38–84*).	Clinical decisions are re-evaluated from patient perspectives	Shared value	Social meaning
During EBM guideline dissemination sessions and clinical training meetings, hospital instructors repeatedly instruct physicians to prioritize patient-centered outcomes when evaluating treatment options (MC-D 3, 5–21).	Patient-centered evaluation criteria are reinforced through formal training and guideline communication	Shared value	Social meaning
“*EBM is an unchangeable trend for medical development, especially in advanced countries*”; “*EBM is continuously developed and has become a hot issue today*” (*BISE 2, 161–180*).	Keep pace with the prevailing trend of medical development	Trendsetter	Social identity
“*EBM provides a novel way of learning*” (*BISE 2, 161–180*).	Embrace innovative learning approaches brought by EBM	Trendsetter	Social identity
“*I project an enterprising image to my colleagues because I devote myself to EBM research*” (*AIJE 4, 63–105*).	EBM involvement projects an enterprising professional image	Pride	Social identity
“*When I provide evidence-based explanations to my patients, they express sincere trust and praise me as a professional doctor*” (*AIJE 1, 36–75*).	Evidence-based practice strengthens professional recognition from patients	Pride	Social identity
During annual performance appraisal processes, physicians' participation in EBM projects is recorded in official evaluation systems and explicitly considered in promotion and professional development decisions (ADS-D 2, 18–60)	EBM engagement is institutionalized as an evaluative criterion in career advancement systems	Pride	Social identity
“*When I come up against problems in my clinical practice, EBM provides suggestions for solutions to the problems… It can promote my desire to learn*” (*AIJE 11, 120–157*).	EBM stimulates motivation for continuous professional learning	Learning promotion	Interpretive infusion
“*Before I came into contact with the EBM system, I had less motivation to learn. Now I can actively retrieve some interesting literature to learn about recent research since I know where the knowledge is to be found*” (*AIJE 11, 120–157*).	Take initiative to explore new research literature	Learning promotion	Interpretive infusion
“*Some patients who possess plentiful medical knowledge question me about more complex problems. Sometimes I feel the pressure from patients' appetite for knowledge*” (*AIJE 11, 120–157*).	Feel learning pressure brought by patients' inquiries	Learning promotion	Interpretive infusion
EBM databases are systematically integrated into daily clinical workflow as a mandatory reference step in diagnostic and treatment processes, shaping physicians' sequential decision-making routines (IR-D 11, 120–157).	EBM becomes structurally embedded into the clinical workflow as a required decision reference point	Learning promotion	Interpretive infusion
“*Since I participated in the EBM training courses, my clinical behavior has become more careful. I always retrieve more evidence to support my decisions*” (*AIS 4, 38–62*).	Perform clinical work more cautiously with evidence support	Attitude change	Interpretive infusion
“*Sometimes I find some excellent studies that conflict with my previous clinical experience; then I carefully examine the new evidence and modify the previous treatment*” (*AISE 7, 35–61*).	Adjust treatment plans based on new evidence	Attitude change	Interpretive infusion
“*EBM realization continuously makes me pursue patient care, which is really good for patients*” (*AISE 7, 35–61*).	EBM reinforces commitment to patient-centered care	Attitude change	Interpretive infusion
“*After the EBM realization, the chances of cross-functional learning and discussions are increasing*” (*AIJE 12, 94–158*); “*Some physicians kindly and enthusiastically post their retrieving outcomes*” (*AIJE 12, 94–158*).	Cross-department discussions and evidence sharing become more frequent	Interaction network formation	Propagative infusion
“*I am glad to see patients' growth. This can push me to move forward*” (*AISE 3, 79–124*).	Patients' positive progress motivates continued engagement in EBM practice	Interaction network formation	Propagative infusion
“*There really exists such an EBM study for the issue. This is so interesting and unbelievable*”; “*The EBM realization changes my focus and skill of reading medical literature*” (*AIS 7, 63–129*).	EBM reshapes approaches to reading and interpreting medical literature	Spillover effect	Propagative infusion
“*The EBM training courses are usually combined with problem-based Learning (PBL)*” (*AIS 7, 63–129*). “*This can enhance the teaching performance for PBL*” (*AISE 4, 18–25*).	EBM-based learning approaches improve PBL teaching practices	Spillover effect	Propagative infusion
In morning clinical teaching rounds, senior physicians project EBM evidence on screens and require trainees to jointly interpret research findings before making clinical recommendations (MC-D 4, 12–38).	EBM evidence is formally embedded into structured clinical teaching and collective interpretation sessions	Spillover effect	Propagative infusion

### 3.5 Reliability and validity

To enhance the reliability and validity of the qualitative findings, the study followed standard procedures for exploratory case study research (Yin, 2014). Construct validity was strengthened through data triangulation across multiple qualitative sources. Specifically, in-depth interviews mainly captured physicians' personal experiences, interpretations, and evolving understandings of the EBM system. Participant observation yielded contextual insights into real interaction practices and behavioral changes amid EBM implementation. Focus group discussions and regular meetings helped identify shared viewpoints and the construction of collective meaning among participants. Documentary materials and archival records supplemented contextual background and cross-checked the consistency of emerging findings.

To ensure analytical rigor, empirical evidence from different sources was continuously compared and verified throughout the iterative coding and analysis process. Major findings and interpretive patterns were repeatedly cross-checked across multiple independent sources to ensure coherence, consistency, and credibility. In addition, participant member checking, iterative comparison, and ongoing discussions during data analysis were employed to further strengthen the trustworthiness and interpretive validity of the study findings. Moreover, as the researchers participated in EBM consulting and on-site observation, prolonged field engagement may give rise to researcher influence and interpretive bias. To minimize such risks, triangulation across multiple empirical sources, iterative comparison, and continuous analytical discussions were consistently applied throughout the research process to maintain reflexivity and analytical rigor.

## 4 Findings: a process model of psychological acceptance

In this study, diffusion denotes the broader dissemination of technology-related meanings and practices across users and professional contexts. Infusion denotes the deeper incorporation of the technology into established professional practices and activities. Based on temporal patterns identified across users' experiential accounts throughout the EBM implementation process, the actions involved in using the EBM service system can be analytically grouped into three behavioral phases (Phase 1–Phase 3). The temporal ordering of these phases was informed by patterns across empirical data collected at different stages of EBM implementation, including participants' accounts, repeated interviews, observations, and documentary materials. Together, the three phases reflect how users' understanding, interpretation, and engagement with the EBM system evolved throughout the implementation process. Phase 1 captures users' initial evaluations grounded in cognitive and psychological benefits associated with functionality and professional support. As users accumulate experience and interaction with the system, Phase 2 reflects the gradual formation of shared meanings and identity-related interpretations through repeated communication and collective understanding among practitioners. Building on these stabilized meanings and identities, Phase 3 represents the deeper infusion of EBM into everyday professional practices, through which technology-related meanings and practices generate broader behavioral effects and diffuse across professional activities and interaction networks.

Inductive analysis further reveals six types of symbolic meanings associated with these usage behaviors: Cognitive benefits, psychological benefits, social meaning, social identity, interpretive infusion and propagative infusion. These findings are summarized in Table 3. The diffusion process of technology-based service innovation is shown in Figure 1. Drawing on inductive coding, the symbolic dimensions embedded in users' system-use behaviors are elaborated in the sections that follow.

**Table 3:** The constructs of the process model of psychological acceptance.

Construct	Definition/sub-construct	Sample data quotations	Relevant literature
Cognitive benefit	Perceived benefit of concrete function and instrumental usefulness by the innovation, concerning support, efficiency, and effectiveness	“*Now, I clearly know how to acquire medical knowledge since the system acts as a learning assistan*t” (AISE 1, 90–154). “*Even a daily and ordinary issue is investigated by a serious experimental design and subsequently formed as valuable evidence… It really breaks my traditional viewpoints on research*” (AISE 8, 42–64).	Acheampong and Cugurullo, 2019; Tang and Son, 2025
Psychological benefit	Perceived benefit of positive emotional state by the innovation, concerning confidence, enjoyment, and attachment	“*When my medical knowledge is continuously updated through EBM learning, I actually feel confident in clinical decisions and patient care*” (AIJE 6, 47–87). “*I can immediately solve some clinical problems, thanks to EBM learning*” (AISE 5, 17–39).	Bui et al., 2025; Chang and Suh, 2025; Naveen et al., 2025; Shi et al., 2025
Social meaning	An intangible symbolic context through which users engage with an innovation, shaped by shared language and shared values	“*Before the EBM realization, almost every physician had his own opinions and hence we frequently had disputes on some issue*s” (AIS 11, 81–135). “*The best evidence is not the best solution for patients*” (CIJE 1, 38–84)	Brill et al., 2025; Naeem et al., 2025
Social identity	An intangible symbolic self-definition through which users position themselves in relation to an innovation, symbolically expressed through trendsetter and pride	“*When I provide evidence-based explanations to my patients, they express sincere trust and praise me as a professional doctor*” (AIJE 1, 36–75). “*EBM provides a novel way of learning*” (BISE 2, 161–180).	Oh et al., 2023; Shao, 2025
Interpretive infusion	An interpretive infusion process of users while accepting the innovation, through which symbolic meanings are cognitively integrated, concerning learning promotion and attitude change	“*EBM realization continuously makes me pursue patient care, which is really good for patients*” (AISE 7, 35–61). EBM databases are systematically integrated into daily clinical workflow as a mandatory reference step in diagnostic and treatment processes, shaping physicians' sequential decision-making routines (IR-D 11, 120–157).	Lai and Hung, 2025; Kim and Jin, 2023
Propagative infusion	A propagative infusion process of users while accepting the innovation, through which symbolic meanings extend beyond individuals, concerning interaction network formation and spillover circulation	“*After the EBM realization, the chances of cross-functional learning and discussions are increasing*” (AIJE 12, 94–158). In morning clinical teaching rounds, senior physicians project EBM evidence on screens and require trainees to jointly interpret research findings before making clinical recommendations (MC-D 4, 12–38).	Miranda et al., 2024; Haj Khalifa et al., 2024

**Figure 1:**
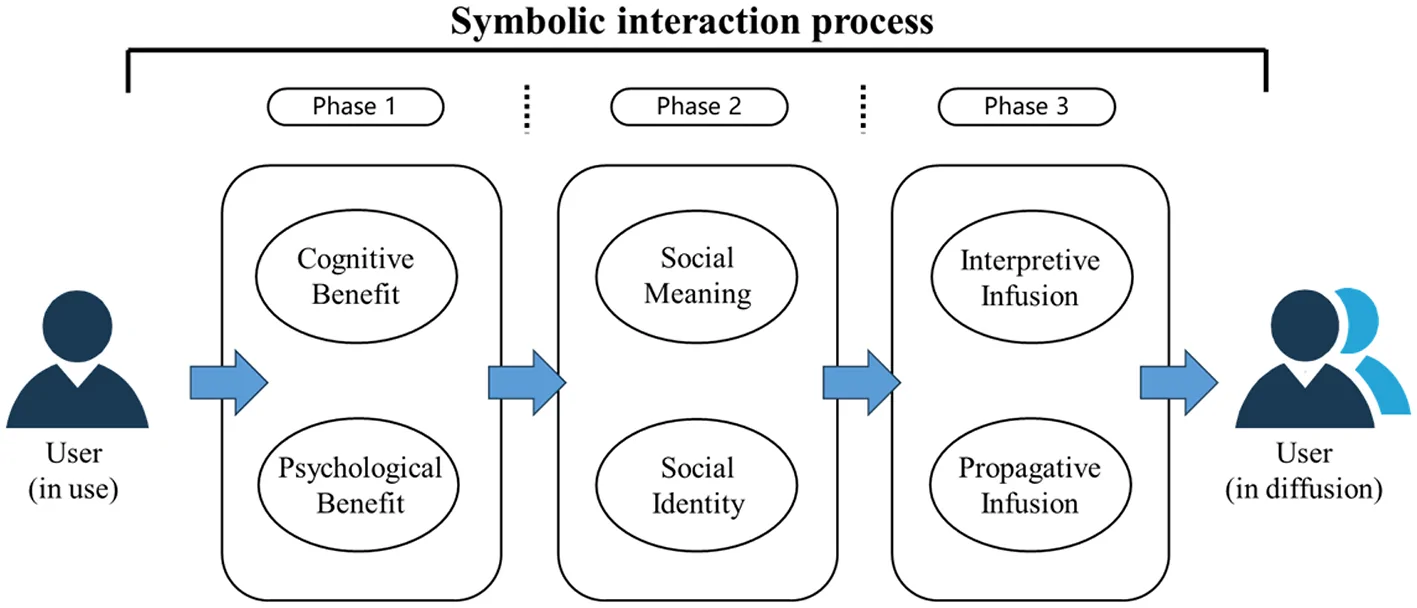
The process model of psychological acceptance in technology-based service innovation.

### 4.1 Phase 1

Phase 1 reflects users' initial encounters with the EBM system. At this stage, acceptance rests primarily on assessments of its functional utility and psychological reassurance. Users focus on whether the system can effectively support clinical learning, decision-making, and routine professional practice, laying an initial evaluative foundation for subsequent meaning construction. This phase focuses on individual-level perceptions arising from initial use of EBM. Specifically, cognitive benefit and psychological benefit represent two distinct dimensions of personal experience. Cognitive benefit emphasizes rational assessment of instrumental functions, whereas psychological benefit captures subjective emotional and psychological responses.

#### 4.1.1 Cognitive benefit

Cognitive benefits capture users' evaluations of an innovation's functional adequacy and instrumental utility. Such evaluations reflect underlying value orientations and cognitive appraisals that shape interaction outcomes with technological systems (Acheampong and Cugurullo, 2019; Tang and Son, 2025). In the study, the symbolism of Cognitive benefit is further expressed as *support, efficiency*, and *effectiveness*. Their meanings are explained below.

##### 4.1.1.1 Support

Physicians feel supported by the EBM system in their quest to acquire, identify, and apply knowledge stored in the EBM databases. Certain conversations are taken as examples: “*Now, I clearly know how to acquire medical knowledge since the system acts as a learning assistant*.” “*Network becomes my textbooks…my teacher*.” and “*There are some good and prefiltered databases in some websites*” (AISE 1, 90–154). When it comes to application, the EBM system mainly supports physicians' clinical practice, academic research, and administrative affairs. Clinical practice includes clinical applications, clinical guidelines, evidence for referring, solutions for referring, clinical decisions, and so on. Administrative affairs include applications for medical affairs, patient education, dissemination of health education, defense for Bureau of National Health Insurance and evaluation and accreditation of hospitals. The EBM system plays a guiding role by providing a platform to search for knowledge, guidelines of literature appraisal, appraisal of clinical application, reading skill, learning guidelines and construction of clinical guidelines. These experiences suggest that EBM is not merely perceived as a technological tool for information retrieval, but is progressively interpreted as an integrated and authoritative learning infrastructure embedded within physicians' professional knowledge acquisition processes.

##### 4.1.1.2 Efficiency

The EBM system efficiently assists users in retrieving medical knowledge, solving problems, and appraising literature. The reason it improves efficiency is that it provides a systematic approach for physicians to learn medical knowledge. Certain quotations reveal the meaning of “efficiency.” “*EBM, just like an assistant, helps me to solve some clinical problems in time*” (AIJE 3, 50–84); “*Sometimes I meet unfamiliar clinical problems questioned by some patients, and then I immediately question EBM and it quickly responds*” (AIS 2, 77–110); “*Some powerful search engines can rank the papers you retrieve… Thus, this saves more time for you*” and “*Since I touch the systematic learning way, I can readily filter papers and efficiently appraise papers*” (CIJE 1, 62–94). These quotes prove that the systematic learning that EBM provides makes it an efficient assistant for retrieving knowledge and solving problems. By reducing the time and effort required to locate and evaluate relevant evidence, EBM enables physicians to make evidence-informed decisions more efficiently in everyday clinical practice.

##### 4.1.1.3 Effectiveness

The effectiveness of EBM databases can be evaluated via three aspects: breadth, depth, and correctness. The three main features of “breadth” are diversity, internationalization, and updating. The physicians consistently mentioned that the diversity of medical issues that the EBM database caters to, broadens their research view. A physician said, “*Even a daily and ordinary issue is investigated by a serious experimental design and subsequently formed as valuable evidence… It really breaks my traditional viewpoints on research*” (AISE 8, 42–64). Internationalization is another critical feature of the EBM database. Research from all over the world can be aggregated on the same platform through the internet. Since it also has information across time, it shows the histories of some issues. Therefore, the medical knowledge is continuously updated and presented in the EBM databases.

When referring to depth, the literature stored in the EBM database is professional and systematic knowledge refined through a rigorous research method. A senior physician said: “*Most of the published papers in the EBM databases possess a rigorous and complete experimental design. By understanding the experimental steps and protocols, the research result is valuable for supporting my clinical decision*” (AISE 5, 29–59). A junior physician said: “*I regard EBM as a professional assistant because it is based on a rigorous and systematic methodology*” (BISE 3, 19–68). Since the research method is rigorous and systematic, the EBM databases can be said to maintain a high level of knowledge depth and methodological credibility. Through repeated interaction with rigorously validated evidence, physicians increasingly interpret EBM as a professionally legitimate and trustworthy basis for clinical judgment.

Finally, while speaking of correctness, the content of the EBM databases is viewed to be objective and valid, since the research of the EBM papers is more scientific and systematic. Some quotes to support it are as follows. “*I think EBM can provide objective and correct solutions for some clinical problems*” (AIS 9, 120–153). “*When I come against unfamiliar clinical problems, the first step I take is to retrieve it* (EBM database), “*This is because the EBM papers have high validity*.” “*If you refer to EBM papers, your solutions are viewed as more standard and correct*.” (BISE 4, 63–101). In addition, as a physician said, “*Some databases, for example, Cochrane library, further provide a prefiltered literature*” (AIJE 10, 82–146). Thus, a prefiltered database increasingly enhances the quality of EBM.

#### 4.1.2 Psychological benefit

Psychological benefits denote the emotional meanings users construct through interaction with an innovation. These benefits are largely hedonic, emerging as technology use generates positive affective experiences and alleviates negative emotions (Naveen et al., 2025). Beyond usability or functionality, psychological benefit represents a subjective sense of experiential meaning and psychological fulfillment shaped through ongoing interaction (Shi et al., 2025). Technologies that convey a stronger sense of perceived presence are therefore more likely to satisfy users' needs for psychological connection (Bui et al., 2025; Chang and Suh, 2025). In the study, the symbolism of psychological benefit includes *confidence, enjoyment*, and *attachment*. Their meanings are explained below.

##### 4.1.2.1 Confidence

The EBM realization can improve confidence of physicians in their clinical practice. Here are some conversation excerpts that voice the viewpoint. “When my medical knowledge is continuously updated through EBM learning, I actually feel confident in clinical decisions and patient care.” “*Through retrieving the EBM papers, I can follow new evidence and medical trends and hence enhance my professional confidence*” (AIJE 6, 47–87). “*I can immediately solve some clinical problems, thanks to EBM learning*.” “*Since the evidence is rigorously derived by researchers, I have more confidence in applying the evidence to my patients*” (AISE 5, 17–39). Thus, physicians feel more confident in their clinical practice after they retrieve and appraise relevant EBM studies. These experiences suggest that EBM-related evidence is gradually internalized as a trusted foundation for professional judgment. Over time, repeated engagement with rigorously validated knowledge strengthens physicians' trust in evidence-based approaches and reinforces their psychological acceptance of EBM.

##### 4.1.2.2 Enjoyment

The feeling of enjoyment mainly comes from finding solutions to some clinical problems. “*I feel so happy to find the solutions to my problems*” (AIJE 7, 115–129). Additionally, one physician said, “*When I browse EBM websites, this is as if I travel in a knowledge palace*” (AIS 5, 25–58). Another physician said, “*To my surprise, I find this case interesting and enjoyable!*” (AISE 2, 63–81). These experiences suggest that interaction with EBM extends beyond functional usefulness and becomes a source of enjoyment in professional learning. As physicians repeatedly explore and apply evidence-based knowledge, the learning process itself becomes associated with feelings of satisfaction, curiosity, and accomplishment.

##### 4.1.2.3 Attachment

After the EBM realization, physicians gradually come to rely on EBM research. Furthermore, they embody the EBM research. Several conversations excerpts support this, for example: “*When I come upon any clinical problem, the first step is to retrieve EBM research… In daily work, I cannot do without EBM*;” “*It is necessary for me to use EBM tools since it provides clinical suggestions*;” and “*I have a high tendency of usage because I need it*” (BISE 1, 59–82). Due to necessity and importance of EBM, the physicians have a high tendency to use and frequency of usage. As one physician said, “*I cannot do without EBM*” (AIS 14, 19–46). Therefore, attachment is reflected in physicians' perception of EBM as a valuable and supportive professional resource. Physicians frequently expressed appreciation for having access to EBM and increasingly regarded it as a trusted companion in their clinical work. As their engagement with the system deepened, EBM became embedded in their professional routines and was viewed as a natural part of everyday clinical practice rather than merely an optional technological tool.

### 4.2 Phase 2

Phase 2 reflects the gradual expansion of acceptance from individual evaluations toward socially shared interpretations and identity-related understandings. As interaction with the EBM system continues, repeated communication and collective understanding among practitioners increasingly shape how the innovation is interpreted, legitimized, and integrated into professional practice. This phase shifts from individual judgment to group-level interactions. Specifically, social meaning and social identity represent two distinct dimensions of social interpretation. Social meaning emphasizes collectively shared understandings surrounding the EBM system, whereas social identity highlights group belonging and role recognition derived from EBM engagement.

#### 4.2.1 Social meaning

Social meaning refers to a shared symbolic context through which users interpret and relate to emerging technologies in digitally mediated environments. That meanings in digital contexts are socially constructed through symbolic interaction rather than embedded in technical artifacts (Brill et al., 2025). Accordingly, users rely on socially shared symbols and cues to assign meaning and guide their engagement with digital artifacts (Naeem et al., 2025). In the study, the symbolism of social meaning is through *shared language and shared value*. Their meanings are explained below.

##### 4.2.1.1 Shared language

The EBM realization results in a shared language for physicians. “*Before the EBM realization, almost every physician had his own opinions and hence we frequently had disputes on some issues*.” “*We have no idea about the validity of some literature before EBM realization. But now we can reach a consensus by following EBM criteria*” (AIS 11, 81–135). “*Physicians and clinical teams jointly discuss and establish standardized criteria for evaluating clinical evidence during EBM implementation meetings*” (OD1 1, 5–27). “*In multidisciplinary case discussions, physicians with different specialties often present divergent treatment opinions, which are ultimately reconciled by referring to shared EBM guidelines and standardized clinical protocols*” (PRO-D 2, 4–18). These accounts suggest that EBM introduces a shared evaluative language through which physicians increasingly interpret clinical issues using common standards and references, thereby reducing interpretive divergence and facilitating the development of shared meanings. From the interview transcripts we understand that this is because EBM realization provides the following: criteria of common agreement, criteria of literature appraisal, standardized process of research presentation, and standardized process of literature discussion.

##### 4.2.1.2 Shared value

The EBM realization can enhance physician–patient communication. “*EBM also teaches me to focus on patient value*.” “*The best evidence is not the best solution for patients*” (CIJE 1, 38–84). “*During EBM guideline dissemination sessions and clinical training meetings, hospital instructors repeatedly instruct physicians to prioritize patient-centered outcomes when evaluating treatment options*” (MC-D 3, 5–21). Thus, the physicians contended that they should reserve patient autonomy and not make decisions for them. Since the EBM realization also enables patients/external customers to acquire medical knowledge, it reduced asymmetric information. This is helpful for patients to communicate better with physicians. As for physicians, they must suggest evidence-based solutions to the patients to avoid dispute. Due to improvements in physician–patient communications, physicians can gain the trust of patients and their families thus improving physician–patient relationship. These accounts suggest that EBM not only improves physician–patient interaction but also gradually reorients clinical value structures toward patient-centered principles, fostering a shared orientation toward patient value in clinical decision-making.

#### 4.2.2 Social identity

Social identity refers to users' perceived social self-definition formed through engagement with an innovation, reflecting how individuals position themselves and are recognized within a social context. Innovations can function as symbolic resources for identity construction by enhancing status, image, social roles, and a sense of belonging, as users seek social recognition and affirmation (Oh et al., 2023; Shao, 2025). In the study, the symbolism of social identity manifests in the form of *trendsetter* and *pride*. Their meanings are explained below.

##### 4.2.2.1 Trendsetter

The medical knowledge in the EBM databases is continuously updated and distributed. If physicians are not privy to the latest knowledge in their fields, their knowledge will be considered out-of-date. “*EBM is an unchangeable trend for medical development, especially in advanced countries*.” “*EBM provides a novel way of learning*.” “*EBM is continuously developed and has become a hot issue today*” (BISE 2, 161–180). These conversations reveal that the EBM system enables physicians to stay up to date with evolving medical trends, which in turn is experienced as a sense of being “fashionable” or “trendy” in their professional practice. Such experiences suggest that engagement with EBM enables physicians to position themselves as early adopters of emerging medical practices, thereby signaling professional progressiveness and alignment with evolving medical trends.

##### 4.2.2.2 Pride

The physicians interviewed were either leaders or key seeded teachers involved in the implementation of EBM. Observations indicated that these physicians experienced a sense of pride when reflecting on how engagement with EBM enhanced their professional image and led them to be perceived by peers as proactive and enterprising. One physician noted, “*I project an enterprising image to my colleagues because I devote myself to EBM research*” (AIJE 4, 63–105). Another remarked, “*When I provide evidence-based explanations to my patients, they express sincere trust and praise me as a professional doctor*” (AIJE 1, 36–75). “*During annual performance appraisal processes, physicians' participation in EBM projects is recorded in official evaluation systems and explicitly considered in promotion and professional development decisions*” (ADS-D 2, 18–60). Although this sense of pride was not always explicitly articulated, it was consistently inferred from the narratives and conversational cues expressed during the interviews. Positive recognition from colleagues and patients contributed to physicians' perceptions of professional legitimacy, fostering a stronger sense of pride in their professional identity.

### 4.3 Phase 3

Building on the shared meanings and identity alignment developed in Phase 2, Phase 3 reflects the further infusion of these interpretations into everyday professional activities and interaction practices. At this stage, users' accumulated interpretations and identity alignment increasingly translate into behavioral influence, knowledge dissemination, and broader diffusion effects across professional interaction networks. This phase concerns the long-term impacts of EBM across professional networks. Specifically, interpretive infusion and propagative infusion represent two distinct dimensions of post-adoption diffusion. Interpretive infusion describes internal changes in personal cognition and practice, whereas propagative infusion embodies external diffusion and interactive effects within the professional community.

#### 4.3.1 Interpretive infusion

Interpretive infusion refers to the process through which users cognitively integrate the symbolic meanings of an innovation into their understanding and evaluation of its use. Through ongoing sensemaking and learning, users interpret the innovation via shared cognitive frames and communicative cues, which shape their attitudes and evaluative orientations toward the technology (Lai and Hung, 2025; Kim and Jin, 2023). In the study, the symbolism of interpretive infusion is through *learning promotion and attitude change*. Their meanings are explained below.

##### 4.3.1.1 Learning promotion

To support the need for learning promotion, we heard several conversations around why physicians need to learn about EBM. “*When I come up against problems in my clinical practice, EBM provides suggestions for solutions to the problems… It can promote my desire to learn*.” “*Before I came into contact with the EBM system, I had less motivation to learn. Now I can actively retrieve some interesting literature to learn about recent research since I know where the knowledge is to be found*.” “*Some patients who possess plentiful medical knowledge question me about more complex problems. Sometimes I feel the pressure from patients' appetite for knowledge*” (AIJE 11, 120–157). “*EBM databases are systematically integrated into daily clinical workflow as a mandatory reference step in diagnostic and treatment processes, shaping physicians' sequential decision-making routines*” (IR-D 11, 120–157). The findings suggest that engagement with EBM not only supports clinical problem-solving but also stimulates physicians' intrinsic motivation to engage in continuous learning, thereby embedding evidence-based inquiry into their ongoing knowledge-seeking behavior.

##### 4.3.1.2 Attitude change

One of the most important changes the physicians experienced is that the EBM realization fosters their rigorous and cautious attitude toward learning. There are three meanings to be discussed. First, the attitude is introspective. Certain opinions the physicians have voiced are, “*Since I participated in the EBM training courses, my clinical behavior has become more careful. I always retrieve more evidence to support my decisions*” (AIS 4, 38–62). “*Sometimes I find some excellent studies that conflict with my previous clinical experience; then I carefully examine the new evidence and modify the previous treatment*.” and “*EBM realization continuously makes me pursue patient care, which is really good for patients*” (AISE 7, 35–61). The introspective attitude includes rigorous and deep learning, self-examination, and emphasis on patient value. Second, it is a continuous learning attitude. Due to the internet, knowledge is distributed and shared more easily. Physicians must continuously update their medical knowledge to provide better patient care. When the EBM concept is gradually embedded into physicians' mindset, physicians imperceptibly show their active and voluntary learning attitude. Third, there's a change in the physicians' practicing attitude for clinical problems. They give emphasis to a scientific and objective way of learning and a rigorous process of clinical experiment and verification after clinical application. In sum, these attitudinal changes indicate that EBM gradually reshapes physicians' professional mindset. Rather than relying primarily on experience or intuition, physicians increasingly adopt continuous learning, critical inquiry, and evidence-based evaluation as guiding principles for clinical practice.

#### 4.3.2 Propagative infusion

Propagative infusion refers to the process through which symbolic meanings linked to an innovation are disseminated through social interactions and network connections, facilitating broader circulation and reinforcement of interpretive frames. This aligns with research on how information spreads through social networks and word-of-mouth dynamics, demonstrating that both direct and indirect social ties influence how innovations are discussed and propagated across communities (Miranda et al., 2024; Haj Khalifa et al., 2024). In the study, the symbolism of propagative infusion is through *interaction network formation* and *spillover effect*. Their meanings are explained below.

##### 4.3.2.1 Interaction network formation

The learning by EBM realization is interactive. Once any researcher is faced with a confusing problem in literature, he/she can reach out to the corresponding authors via the internet. Some conversations show that there are some other interactions among the physicians. “*After the EBM realization, the chances of cross-functional learning and discussions are increasing*.” “*Some physicians kindly and enthusiastically post their retrieving outcomes*.” (AIJE 12, 94–158). The EBM realization results in cross-functional learning and discussions via knowledge sharing and group learning. In addition, since learning through the EBM realization is scientific and objective, it facilitates knowledge impartation from master to apprentice. The last interactive condition, exists between physicians and patients. Due to the democratization of medical knowledge by the EBM realization, a positive growth of knowledge in patients is expected. A physician said, “*I am glad to see patients' growth. This can push me to move forward*” (AISE 3, 79–124). In sum, these interactional processes suggest that EBM contributes to the formation of broader interaction networks among physicians. These networks provide channels through which evidence-based knowledge and learning practices can be continuously exchanged and reinforced.

##### 4.3.2.2 Spillover effect

The EBM realization also has spillover effects for physicians. “*There really exists such an EBM study for the issue. This is so interesting and unbelievable*.” “*The EBM realization changes my focus and skill of reading medical literature*.” “*The EBM training courses are usually combined with problem-based Learning (PBL)*” (AIS 7, 63–129). “*In morning clinical teaching rounds, senior physicians project EBM evidence on screens and require trainees to jointly interpret research findings before making clinical recommendations*” (MC-D 4, 12–38). “*This can enhance the teaching performance for PBL*” (AISE 4, 18–25). The EBM realization eventually broadens the views of research, improves learning skills, and leads to a new way of teaching. Some other opinions contended that EBM realization makes users understand recent research, expand their field of learning, and promote medical issues. In sum, these spillover effects indicate that engagement with EBM extends beyond clinical practice and progressively enables the transfer of evidence-based learning principles into research cognition, pedagogical practices, and broader professional and everyday behavioral contexts.

## 5 Discussions

This study develops an integrative process model of technology-based service innovation (Figure 1) that reconceptualizes diffusion not as a discrete adoption decision, but as a staged process of meaning construction and stabilization in use. Departing from dominant diffusion and technology acceptance perspectives that frame adoption primarily as an outcome of rational evaluations of technological attributes (Rogers, 2003; Davis, 1989; de Brauwer, 2024), the model foregrounds how acceptance unfolds through successive meaning-related phases. In Phase 1, users' engagement with technology-based services is initially anchored in cognitive benefits, such as perceived usefulness and functional relevance, which provide the evaluative basis for early judgments (Marian et al., 2025). These cognitive assessments subsequently translate into psychological benefits, including feelings of reassurance, confidence, and reduced uncertainty, as users begin to internalize the technology within their subjective evaluative frames (Liu N., 2025). Thus, cognitive and psychological benefits constitute the foundational evaluative meanings that enable subsequent processes of interpretive integration and meaning internalization. This sequential shift from functional evaluation to internalized psychological benefit consistently appears across interview narratives, where practitioners repeatedly described moving from initial tool-focused assessment to gradual subjective acceptance of the service's professional value.

However, empirical findings suggest that positive evaluation does not automatically translate into sustained use. Phase 2 marks a critical transition in which users move beyond individual assessment toward constructing social meaning. Shared values and interpretive language forged through clinical ward rounds, multidisciplinary dialogue, and regular departmental case discussions begin to shape how the innovation is understood and enacted in everyday clinical work. Cognitive and psychological benefits provide the foundation for the emergence of social meaning and social identity. While cognitive benefits enable physicians to develop shared understandings of the value and role of EBM in clinical practice, psychological benefits strengthen identification with evidence-based practice as an appropriate and valued approach to clinical work (Li et al., 2025; Heiskari et al., 2025). Through repeated interaction, the principles embedded in EBM gradually become integrated into physicians' understandings of professional responsibility, clinical judgment, and appropriate practice (Hu et al., 2024). Social meaning and social identity jointly transform EBM from an externally evaluated technology into a professionally embedded mode of clinical practice. This perspective also helps explain an important discrepancy between behavioral compliance and psychological acceptance. Consistent with prior research on professional identity and technology use, excessive rationalization may generate resistance when technological systems are perceived as incongruent with established professional meanings and practices (Elgin and Elgin, 2024; Pardoux and Kerasidou, 2025; Ackerhans et al., 2025). The findings of this study further suggest that constrained acceptance stems less from technological functionality than from the failure to establish shared professional meanings around technology use. When physicians perceived a disconnect between the logic embedded in the EBM system and established clinical routines or professional conventions, they found it difficult to make sense of the system within their existing professional context. In healthcare settings, where professional norms strongly influence practice, the development of shared meanings becomes critical for aligning technological innovations with professional expectations and facilitating sustained diffusion.

Phase 3 extends diffusion beyond routinized use to behavioral infusion, as socially constructed meanings and identities begin to translate into observable action patterns. This perspective builds on post-adoption research on continuance, routinization, and infusion, which has primarily examined how technology use is sustained, becomes habitual, or becomes deeply embedded in users' practices after initial adoption (Hassandoust et al., 2024; Schwarz and Chin, 2024). In contrast, the present study conceptualizes infusion as a meaning-based process through which users progressively incorporate the technology into their professional identities and practices. Shared social meanings established earlier continue to operate through interpretive infusion, promoting ongoing learning and attitude change that deepen users' understanding of the innovation in practice (Agarwal et al., 2026). Interpretive infusion works as a diffusion mechanism by reducing the cognitive and symbolic distance between early users and potential adopters. Moreover, this identity positioning evokes positive emotions, particularly pride, which motivates users to make their practices more visible and exemplary. Thus, interpretive infusion differs from continuance or routinization in that it concerns how users reconstruct the meaning and identity significance of technology through continued engagement, rather than simply whether technology use is sustained or becomes habitual. As a result, behavioral spillovers emerge not through abstract knowledge transfer, but through observable shifts in professional conduct and interaction patterns that gradually reinforce technology use within professional communities (Blair et al., 2019; Kim et al., 2025). As these practices become visible and reproducible, propagative infusion captures how technology-related meanings and practices extend beyond individual users and enter broader professional interactions. Immersive and self-reinforcing usage patterns render visible practices salient and reproducible, facilitating the continued stabilization and diffusion of technology-based service innovation across broader professional contexts (Huang et al., 2025). In Phase 3, interpretive and propagative infusion operate together to sustain diffusion, allowing meanings, identities, and patterns of action to be continually reproduced and extended. This reproduction was facilitated through localized behavioral spillover, as early adopters' visible use of EBM during routine clinical decision-making provided interpretive cues that enabled peers to construct similar understandings and gradually incorporate EBM into their own clinical practice.

In sum, the study advances existing understanding of technology-based service innovation by conceptualizing psychological acceptance as a process of symbolic stabilization that unfolds through ongoing interaction. Importantly, the temporal progression identified in this study should not be interpreted as a fixed sequence that every individual user necessarily follows. Rather, the three phases represent analytically distinct forms of meaning construction that may overlap or recur across users as diffusion unfolds. The emergence of a later phase does not eliminate earlier forms of meaning construction. Earlier forms may persist and coexist with later ones. Over time, however, the characteristics of later phases become increasingly prominent in shaping how meanings are interpreted, negotiated, and reconstructed. The model moves beyond viewing diffusion simply as a consequence of technological attributes or adoption decisions (Mele et al., 2019) by highlighting how meanings, identities, and behavioral patterns are progressively constructed, adjusted, and propagated through continued technology use. This perspective suggests that the functional advantages of rationalized technologies alone do not fully explain their diffusion, as continued use also depends on how technological rationality becomes meaningful and compatible with users' professional contexts (Chen et al., 2020). By integrating symbolic benefits, social meanings, interpretive infusion, and identity-related behavioral influence within a unified process framework, the study provides a process-oriented explanation of how psychological acceptance emerges, evolves, and diffuses over time.

## 6 Conclusions

This study explains how psychological acceptance of technology-based service innovation evolves during the diffusion process. Drawing on symbolic interactionism, the findings show that psychological acceptance is not an immediate consequence of adoption but develops through ongoing symbolic interaction and the continuous construction and reinterpretation of meanings. Through these processes, technological rationality and professional identity become progressively reconciled as technology is integrated into everyday practice. Building on these findings, the study contributes to the literature on technology-based service innovation and diffusion in three respects. First, the study advances diffusion theory by moving beyond outcome-oriented models of adoption toward a processual and interpretive account of how technology-based services become stabilized in use. Rather than viewing diffusion as the successful completion of adoption, it conceptualizes diffusion as an ongoing process through which technologies become socially embedded and stabilized in everyday practice. Second, the study develops a process model of psychological acceptance in technology-based service innovation. Existing research has predominantly explained acceptance in terms of adoption decisions, usability evaluations, or interaction experiences. In contrast, the proposed model explains how psychological acceptance emerges and develops through ongoing symbolic interaction, highlighting the role of meaning construction in shaping users' responses to technology throughout the diffusion process. Third, the study reconceptualizes the paradox of rationalization from an adoption to a diffusion mechanism. Rather than viewing the tension between technological objectivity and professional agency as a problem that must be resolved prior to successful adoption, the findings show that it remains an enduring feature of diffusion and is continually negotiated throughout implementation and use. This perspective shifts attention from optimizing technological functionality to understanding how professional legitimacy and technological rationality become aligned during the diffusion process.

From a managerial perspective, the findings suggest that successful diffusion of technology-based service innovation requires more than improving usability, functionality, or performance metrics. Managers and system designers should attend to the social and symbolic conditions under which technologies are introduced and embedded in everyday work practices. In particular, fostering spaces for interaction, dialogue, and peer sensemaking can be as critical as technical training in enabling sustained use. Rather than framing implementation as a top-down process of rational persuasion, organizations should support the co-construction of shared meanings that align technological capabilities with users' professional identities and values. Such an approach not only strengthens continued engagement but also enables positive behavioral spillovers, through which individual adoption becomes collectively reinforced and scaled across the service ecosystem.

Despite these contributions, this study is subject to several limitations that also provide directions for future research. First, the study was conducted within a defined period of EBM implementation and diffusion, which limits its ability to capture how meaning construction and behavioral practices may continue to evolve over a longer period. Future research may adopt a longitudinal design to track changes in the core variables over time and further validate their causal relationships. Second, the empirical data were drawn from professional practitioners in the EBM service context, with the interviewed physicians having extensive EBM experience and serving primarily as EBM team leaders or seeded teachers responsible for promoting EBM. This participant profile may have contributed to the predominantly positive meanings identified in the study and limits the transferability of the findings to less committed, reluctant, or non-adopting users. Future research could explore these different user groups across industries and contexts to assess the boundary conditions of the proposed process model. Third, this research primarily focuses on end users' psychological experience and social-symbolic interaction, while overlooking institutional and governance factors across the service ecosystem. Future research can incorporate multi-level perspectives to explore how external factors moderate the tension between technological rationality and professional identity during diffusion. Fourth, this study primarily focuses on users who have adopted technology-based services and therefore emphasizes the processes through which positive psychological acceptance and symbolic meanings are constructed over time. As a result, negative symbolic interpretations and oppositional meaning structures associated with the technology were not systematically incorporated into the empirical analysis. Future research could further examine how negative symbolic meanings, identity conflicts, and resistant interpretations shape users' attitudes and behavioral responses, thereby enabling a more comprehensive understanding of symbolic meaning construction within diffusion processes.

## Data Availability

The original contributions presented in the study are included in the article/supplementary material, further inquiries can be directed to the corresponding authors.
